# Low-Dose Maintenance Intravenous Iron Therapy Can Prevent Anemia in Children with End-Stage Renal Disease Undergoing Chronic Hemodialysis

**DOI:** 10.1155/2020/3067453

**Published:** 2020-06-01

**Authors:** Cahyani Gita Ambarsari, Partini Pudjiastuti Trihono, Agustina Kadaristiana, Dedi Rachmadi, Murti Andriastuti, Henny Adriani Puspitasari, Taralan Tambunan, Sudung Oloan Pardede, Irawan Mangunatmadja, Eka Laksmi Hidayati

**Affiliations:** ^1^Pediatric Nephrology Division, Department of Child Health, Faculty of Medicine Universitas Indonesia, Cipto Mangunkusumo Hospital, URJT Building 5th Floor, Diponegoro 71, Jakarta Pusat 10430, Indonesia; ^2^Pediatric Nephrology Division, Department of Child Health, Faculty of Medicine Universitas Padjadjaran, Dr. Hasan Sadikin General Hospital, Pasteur 38, Bandung 40161, Indonesia; ^3^Pediatric Hematology-Oncology Division, Department of Child Health, Faculty of Medicine Universitas Indonesia, Cipto Mangunkusumo Hospital, URJT Building 5th Floor, Diponegoro 71, Jakarta Pusat 10430, Indonesia; ^4^Pediatric Neurology Division, Department of Child Health, Faculty of Medicine Universitas Indonesia, Cipto Mangunkusumo Hospital, URJT Building 5th Floor, Diponegoro 71, Jakarta Pusat 10430, Indonesia

## Abstract

Iron deficiency anemia is common in children with end-stage renal disease (ESRD) on long-term hemodialysis receiving erythropoiesis-stimulating agents. One approach to maintain the iron profile and hemoglobin levels is maintenance therapy with regular low doses of intravenous (IV) iron after initial iron repletion therapy; however, evidence for the benefits of this approach is lacking. This study evaluated the effect of IV iron maintenance therapy on anemia in children on regular hemodialysis. This retrospective cohort study included 41 pediatric ESRD patients with normal hemoglobin and iron status who underwent regular hemodialysis at the Pediatric Dialysis Unit of Cipto Mangunkusumo Hospital, Indonesia, between January 2015 and April 2019. Among these, 21 received IV iron maintenance therapy with two doses of 2 mg/kg of IV iron sucrose every 2 weeks (the treatment group) and 20 did not (the comparison group). Changes in hemoglobin and transferrin saturation were assessed after 6 weeks of observation and compared between the two groups. There was a significant reduction in the mean hemoglobin level compared with the baseline level in the comparison group (21 g/L; 95% CI, 9.3–33 g/L; *p*=0.001) but not in the treatment group (0.7 g/L; 95% CI, −6.6–8 g/L; *p*=0.84). The risk of anemia was lower in the treatment group (relative risk = 0.42; 95% CI, 0.22–0.79; *p*=0.003). Although majority of the patients had high baseline ferritin level, this study indicates that in our setting, ferritin may not be a reliable parameter to review the iron status, as it can be affected by chronic inflammation. Hence, the decision to start IV iron maintenance therapy in patients with hyperferritinemia should consider the patient's clinical condition and morbidity. To conclude, the coadministration of IV iron maintenance therapy is beneficial for maintaining hemoglobin levels and preventing anemia in children with ESRD who are undergoing regular hemodialysis, have achieved the target hemoglobin levels, and have normal iron status.

## 1. Introduction

Anemia is a severe complication commonly experienced by patients with end-stage renal disease (ESRD) who undergo regular hemodialysis (HD); in children, its prevalence with a hemoglobin level of <110 g/L has been reported to be over 93% [1]. The Know-Ped CKD study from Korea reported that anemia in pediatric chronic kidney disease (CKD) was associated with poor nutritional status and 72% had iron deficiency [2]. This is devastating because anemia is known to be associated with low quality of life, in addition to high morbidity and mortality [3]. A recent study from our center revealed that most of our pediatric ESRD patients had anemia, leading to cardiac morbidity, and may have contributed to death [4].

The Kidney Disease Improving Global Outcome (KDIGO) and the Renal Association have described two methods of iron therapy commonly used for ESRD patients undergoing HD: iron repletion (an episodic iron therapy administered when the patient experiences iron deficiency) and maintenance therapy (in which regular iron supplementation at a lower dose is administered) [5, 6]. In contrast to the iron repletion method, there is a lack of strong evidence to recommend iron maintenance therapy for this patient population [5]. In addition, there is no clear recommendation on whether erythropoiesis-stimulating agents (ESAs) should be coadministered with iron supplementation in the management of anemia [7], and there is no consensus between nephrologists and hematologists over the use of intravenous (IV) iron therapy [5]. The aim of this study, therefore, was to identify whether IV iron maintenance therapy with ESAs is beneficial for preventing anemia in children with ESRD.

## 2. Materials and Methods

### 2.1. Study Design and Patients

This retrospective cohort study was conducted between January 2015 and April 2019. The inclusion criteria were patients aged <18 years with ESRD who underwent HD for at least 3 months at the Pediatric Dialysis Unit of Cipto Mangunkusumo Hospital and who had normal hemoglobin and iron levels, with ESA therapy at a stable dose (within ±25%), during the month prior to the initial follow-up. The criteria used to define normal hemoglobin levels were those reported by KDIGO: ≥110 g/L for children aged 0.5–5 years, ≥115 g/L for children aged 5–12 years, ≥120 g/L for children aged 12–15 years, ≥120 g/L for girls aged >15 years, and ≥130 g/L for boys aged >15 years [5]. The iron requirement was as recommended by KDIGO for pediatric patients with ESRD who undergo HD: ferritin >100 *μ*/mL and transferrin saturation (TSAT) >20% [5]. The exclusion criteria were as follows: hemolytic anemia, bleeding manifestation, hemoglobinopathy, poor nutrition, severe infection or inflammation, the receipt of red blood cell or whole blood transfusion during the follow-up period, transfer to the adult HD unit, conversion to peritoneal dialysis, or incomplete medical records. Complete demographic and clinical data were obtained from the medical records of the included patients.

The study was approved by the Cipto Mangunkusumo Hospital Institutional Review Board and the Ethics Committee of the Faculty of Medicine, University of Indonesia (KET-566/UN2.F1/ETIK/PPM.00.02/2019). The requirement for patient consent was waived because of the retrospective nature of this study.

Prior to this study, our center had no protocol for anemia management for pediatric HD patients. The eligible patients were retrospectively categorized into two groups: a treatment group that included the patients who received iron maintenance therapy and a comparison group that included the remaining patients. The treatment group patients received 2 mg/kg of IV iron sucrose every 2 weeks for two doses. The iron sucrose was diluted in 0.9% saline solution at a concentration of 1 mg/mL and was administered at a rate of 100 mL/h. The comparison group patients did not receive the iron maintenance therapy. None of the patients received oral iron therapy during the study period.

The follow-up period began when the patients achieved normal hemoglobin levels and iron status and lasted for 6 weeks. During this period, patients in both groups continued to undergo HD according to a constant regimen, in which the duration, blood flow, dialysate flow, ultrafiltration, and heparin dose had remained constant within the previous 1 month. ESAs were administered to all the patients at stable doses beginning at least 1 month prior to the study and continuing until at least the end of the follow-up period.

The primary outcome was the proportion of patients in each group who experienced anemia during the 6-week follow-up period. The secondary outcomes were changes in hemoglobin and TSAT levels. Specifically for the purposes of this study, patients were considered to have iron depletion when their hemoglobin levels were normal for their age, but their TSAT levels were <20%. Iron overload was defined as TSAT levels >50% [8].

### 2.2. Statistical Analysis

Continuous variables are presented as mean values with 95% confidence intervals and categorical variables as proportions and percentages. Data were tested for normality and homogeneity of variance using the coefficient of variation and Levene's test, respectively. Independent continuous data were compared using Student's *t*-test if they had equal variance or Welch's test for unequal variance. Dependent continuous data were compared using the paired *t*-tests. Categorical data were compared using the chi-square test, unless the assumptions for chi-square were not met, in which case Fisher's exact test was used. The mean hemoglobin and TSAT levels at the beginning and end of the study period were compared using two-way repeated-measures ANOVA for both groups. The Bonferroni correction was used to identify the mean change in hemoglobin levels between the beginning and end of the study period in the comparison group. To determine the mean difference in ferritin levels between the groups at the start and end of the study period, a mixed-model analysis was applied because of missing data values.

*p* values <0.05 were considered statistically significant. The statistical analysis was performed using SPSS 24.0 (IBM Corp., Armonk, NY, USA) and GraphPad Prism 8.1.1 (GraphPad Software, San Diego, CA, USA).

## 3. Results

**Figure 1:**
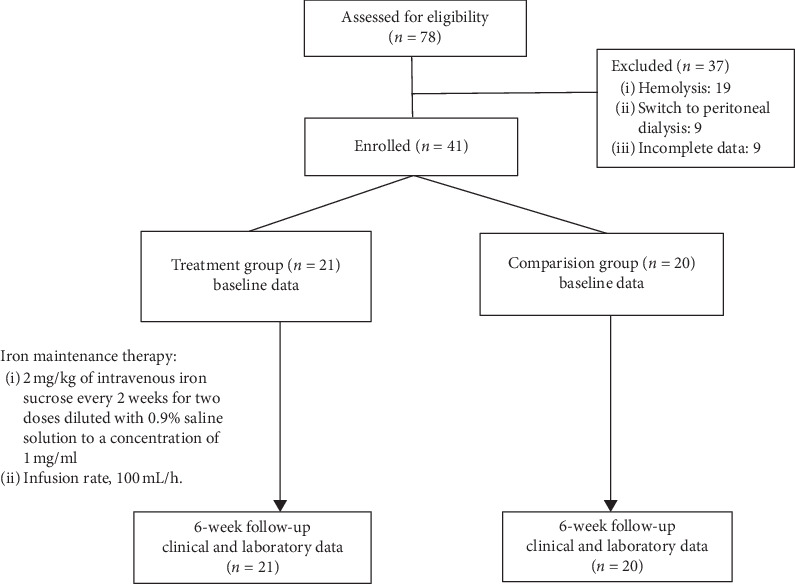
Patient flowchart.

Between January 2015 and May 2019, 78 children with ESRD underwent regular HD with normal hemoglobin and iron status at our Pediatric Dialysis Unit. Among these, 37 were excluded because of hemolysis (*n* = 19), switch to peritoneal dialysis (*n* = 9), or incomplete data (*n* = 9). Hence, the study included 41 pediatric patients (Figure 1), of which 21 received IV iron maintenance therapy (the treatment group), whereas the other 20 patients did not (the comparison group). Apart from serum iron levels, there were no significant differences in the baseline characteristics between the groups. The serum iron levels were significantly lower in the treatment group than in the comparison group, but the serum iron levels in both groups were within the normal limits.

Table 1 summarizes the baseline characteristics of the patients in the two groups. The majority of patients in both groups were boys. The mean ages were 13.5 years (95% CI, 12–15.1 years) for the treatment group and 14.7 years (95% CI, 13.8–15.7 years) for the comparison group. Primary kidney diseases found in the treatment group included renal hypoplasia and dysplasia (10/21, 48%), glomerulopathy (8/21, 38%), and urological disorders (3/21, 14%); in the comparison group they included hypoplasia and dysplasia (8/20, 40%), glomerulopathy (8/20, 40%), and urological disorders (4/20, 20%). The patients' ferritin levels were relatively high, but there was no significant correlation between ferritin levels and nutritional status (*p*=0.45).

**Figure 2:**
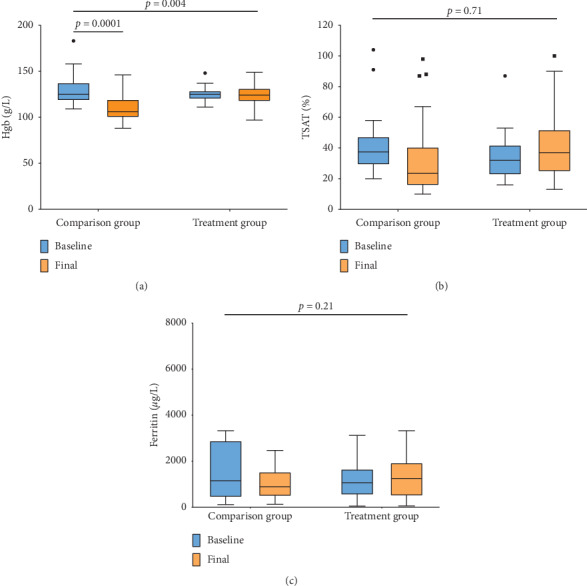
Hemoglobin (Hb), transferrin saturation (TSAT), and ferritin levels at baseline and at the end of the 6-week study period. The horizontal lines indicate the median value, the boxes indicate interquartile range, and the whiskers indicate the minimum-maximum value. The closed circles and squares in the first two figures represent outlying points.

Table 2 summarizes the patients' characteristics at the end of the 6-week period, and Figure 2 shows comparisons between the baseline and final values of hemoglobin, TSAT, and ferritin levels in each group. In the treatment group, hemoglobin levels were maintained within the normal range, with a mean difference between baseline and final hemoglobin levels of 0.7 g/L (95% CI, −6.6 to +8.0 g/L; *p*=0.84). Conversely, the comparison group experienced a significant reduction in hemoglobin levels by 21 g/L (95% CI, 9.3–33 g/L; *p*=0.001). There were no significant changes in TSAT levels, although there was a nonsignificant increase in the treatment group of +7.7% (95% CI, −3.6% to +19%; *p*=0.171) and a nonsignificant decrease in the comparison group of –8.5% (95% CI, −22.9% to +5.9%; *p*=0.23). There were no significant changes in ferritin levels.

Anemia was categorized based on hemoglobin level and age. The risk of anemia was significantly lower for the treatment group than for the comparison group (7/21 [33%] vs. 16/20 [80%]; relative risk = 0.42; 95% CI, 0.22–0.79; *p*=0.003). Six of the treatment group patients (29%) experienced iron overload (TSAT level >50%) although they did not exhibit any symptoms. Iron depletion, defined by normal hemoglobin levels consistent with the patient's age but low TSAT levels, occurred in 2/21 (9.5%) patients in the treatment group and in 3/20 (15%) patients in the comparison group. No patient experienced any side effects of the IV iron therapy on the cardiovascular system or central nervous system, and there were no dermatological, gastrointestinal, neuromuscular, or respiratory side effects, or life-threatening reactions, such as anaphylaxis.

## 4. Discussion

Iron supplementation is known to be beneficial for stimulating erythropoiesis and increasing hemoglobin levels in patients with CKD and for reducing the dose of ESAs required by patients with ESRD who undergo HD [9, 10]. However, there is no consensus on the optimal strategy for iron therapy in children with ESRD who undergo HD [11]. In addition, there is a difference of opinion over the use of iron therapy as a maintenance strategy. KDIGO recommends a loading dose of iron repletion therapy when TSAT ≤20% and ferritin ≤100 *μ*g/mL but has no recommendation for a maintenance dose because of the lack of evidence [5]. Conversely, the Renal Association recommends IV iron maintenance therapy for patients with CKD who receive ESAs and who have achieved the target iron requirement [6]. There are also concerns among clinicians about high-dose iron therapy for HD patients. Based on a retrospective study that involved 117,050 adult patients, therapy involving a high-dose bolus of iron increased the risk of infection-related hospitalization, particularly in patients with central venous catheters; however, this was not observed in patients who received iron as a maintenance dose [12].

The results of the present study support the recommendations issued by the Renal Association on providing iron maintenance therapy. They showed that iron maintenance therapy in children with ESRD who were undergoing HD could reduce the risk of anemia and improve the maintenance of hemoglobin levels. The specific iron supplementation therapy used in the study involved the IV administration of iron sucrose. IV administration is recommended for pediatric patients with ESRD who undergo HD because it has been shown to be more effective than oral treatment for correcting iron depletion, increasing hemoglobin levels, and reducing the need for ESAs [13, 14]. The available forms of IV iron preparations in developed countries are iron dextran, sodium ferric gluconate, iron sucrose, and ferumoxytol. There is a 0.61% risk of anaphylaxis with IV iron dextran therapy, but the risk is reduced to 0.04% with IV sodium ferric gluconate. Iron sucrose was administered to the patients in the present study because it is the only preparation available in Indonesia. It has a similar safety profile to that of IV sodium ferric gluconate [15].

To the best of our knowledge, no previous study has compared the effects of IV iron therapy with and without maintenance dosing on preventing anemia in children who undergo regular HD after iron repletion. However, in a randomized clinical trial, Ruiz-Jaramillo et al. [16] compared two groups of children with ESRD who were undergoing HD and had either absolute or functional iron deficiency. The first group received iron therapy consistent with their ferritin level, starting with a loading dose, followed by weekly maintenance doses until achieving TSAT >50% and ferritin >800 *μ*g/L. The second group received an intermittent dose for 10 weeks. The study revealed that iron maintenance therapy based on serum ferritin levels was better than administering intermittent doses for achieving the desired hemoglobin and ferritin levels, with a smaller risk of iron overload.

In a study by Rottembourg et al. [17], two groups of adult patients with renal failure received either IV iron therapy prior to the initiation of HD or no IV iron therapy. Both groups received 50 mg/week of IV iron therapy during their HD treatment, in addition to ESAs. The hemoglobin and TSAT levels were compared between the initiation of HD and 12 months later. The study results showed that, for ESRD patients undergoing regular HD, IV iron therapy with maintenance dosing administered concomitantly with ESAs improved hemoglobin and TSAT levels, in both the patients who initially had low levels of hemoglobin and/or TSAT and the patients who initially had normal levels of hemoglobin and/or TSAT [17]. Consistent with these findings, our study showed that IV iron therapy with maintenance dosing resulted in similar hemoglobin levels between 125 (121–129) g/L at baseline and 124 (118–131) g/L 6 weeks later, whereas in the comparison group the mean hemoglobin level decreased from 131 (123–140) g/L to 110 (103–118) g/L. However, unlike the results of Rottembourg et al., we did not find a significant change in mean TSAT levels over the follow-up period in either group, although three of the 20 patients in the comparison group experienced iron depletion. This difference between the studies may have been due to the short duration of the present study, which may not have allowed sufficient time for a reduction in TSAT to be observed. A study in an adult population showed that IV iron maintenance therapy with a very low dose (15.56 ± 42.4 mg/week) resulted in a significant reduction in TSAT only after a 3-month observation [18]. Another possible reason for the low TSAT levels despite the elevated ferritin levels may have been the hepcidin-mediated blockade of iron mobilization from the reticuloendothelial system [19].

In a randomized clinical trial that included subjects with similar age characteristics to those in the present study, Goldstein et al. [20] administered one of three doses of IV iron sucrose (0.5, 1.0, or 2.0 mg/kg) to patients aged 2–20 years once every other week for six doses. The maintenance dose of 2 mg/kg/2 weeks achieved a success rate of 43% for maintaining hemoglobin levels of ≥10.5 g/dL and TSAT ≥20%. In comparison, our study included pediatric patients aged <18 years, and there was a higher range of success parameters, defined as normal hemoglobin levels for the patient's age according to the KDIGO recommendations. In our study, IV iron therapy at a dosage of 2 mg/kg/2 weeks was successful in 14 of the 21 patients (67%) in maintaining normal hemoglobin levels (according to the KDIGO recommendations) and TSAT ≥20%. The higher success rate in our study may be explained by differences in study methods. In Goldstein et al.'s study, all the patients received IV iron therapy and the follow-up duration was longer (3 months compared with 6 weeks in our study).

A unique characteristic of our patients was their high baseline serum ferritin levels. This hyperferritinemia may have been resulted from multiple transfusions before HD initiation. The majority of cases referred to our center are in acutely ill condition with severe anemia requiring packed red cell transfusions or have received blood transfusions prior to referral [21, 22]. The pediatric dialysis unit has not been widely available in Indonesia [4]; therefore, referral system has been a major issue, since Indonesia is an archipelago consisting of 17,504 islands [23]. As a result of the complex referral system between islands, most patients have significant clinical deterioration upon admission [24]. This baseline hyperferritinemia is a distinctive challenge at our unit because providing patients with high ferritin levels with IV iron maintenance is controversial. As yet, there is no guideline for determining the upper threshold of ferritin levels in patients with CKD [25]. At our unit, the decision to administer IV iron maintenance therapy in children on regular HD with high ferritin levels was made carefully after considering the benefits of preventing anemia and the risks of iron toxicity. We agree with Kelepouris and Kalantar-Zadeh [26], who have suggested that the decision to administer iron supplementation therapy should be reached by adopting a holistic approach, taking into consideration the patient's comorbidities and the risk of iron deficiency, which may be more harmful than hyperferritinemia.

It is also worth noting that high ferritin concentration does not necessarily result in clinically relevant iron toxicity in patients with CKD [27]. Serum ferritin levels in the range 200–2000 *μ*g/L in patients who undergo HD may be the result of malnutrition-inflammation complex syndrome, i.e., protein-energy malnutrition accompanied by inflammation [28]. In the present study, there was no significant correlation between nutritional status and ferritin levels. When ferritin levels are high, they are not a reliable parameter for evaluating iron status. The serum ferritin level is affected by inflammation and is an acute phase reactant, so it should be interpreted with caution in patients with CKD. Most ESRD patients, including those who undergo chronic HD, have normal marrow iron storage when the serum ferritin level is ≥300 *μ*g/mL [29]. In the present study, 37 of the 41 patients (90%) had catheter access for HD, so it was assumed that the high ferritin levels were due to chronic inflammation as a result of HD catheter usage. However, we did not measure C-reactive protein level as evidence of chronic inflammation or infection because the patients exhibited no symptoms of fever or leukocytosis that indicated a need for laboratory tests of inflammatory markers. Biofilms form on HD catheters, which can induce an inflammatory response; however, there was no fever or leukocytosis, and no positive blood culture results [30].

The Dialysis Patients' Response to IV Iron with Elevated Ferritin (DRIVE) trial [31] reported that IV iron therapy with increased doses of ESAs for patients with high ferritin levels (500–1200 *μ*g/mL) and low TSAT (≤25%) increased short-term hemoglobin levels without any obvious additional side effects compared with those of patients who did not receive IV iron. The findings of this study and of the studies described earlier suggest that a laboratory result showing hyperferritinemia should not restrain physicians from performing optimal anemia management [26]. Since hyperferritinemia may be resulted from inflammation, infection, liver disease, and malignancy, all of which should be investigated [27]. If erythropoietin resistance persists, iron supplementation may be provided.

Because of the limitations of using serum ferritin levels for the assessment of iron status in pediatric ESRD patients with anemia, another test is required to evaluate iron status in red blood cells, the most important aspect of iron homeostasis. A meta-analysis from the UK National Institute for Health and Care Excellence 2016 guidelines [32] reported that percent hypochromic red blood cells (%HRC) and reticulocyte hemoglobin content (CHr) are more sensitive than serum iron for detecting relative iron deficiency, and both parameters are better than ferritin level for evaluating the response to iron therapy. The cutoff points used in these guidelines to diagnose iron deficiency are %HRC >6% and CHr <29 pg [32]. Although these tests are promising, many laboratories, including the laboratory at our hospital, are unable to perform the required analyses. Availability of resources to diagnose and manage complex cases in Indonesia can be limited, therefore, we need to come up with alternative diagnostic tool or regimen. A recently published case from our center also reported the use of alternative medication because the recommended regimen is not available in Indonesia [33].

The present study provides valuable new data on the role of IV iron maintenance therapy on anemia in pediatric patients with ESRD who undergo regular HD. Until now, there has been a lack of evidence for this approach. The study demonstrated the effects of IV iron maintenance therapy in maintaining hemoglobin levels and preventing anemia. It also showed that there is the possibility that pediatric patients may develop iron overload, despite the low doses of IV iron, and that administering IV iron maintenance therapy to pediatric patients undergoing chronic HD who had high ferritin levels did not necessarily result in clinically relevant iron toxicity, so providing iron therapy to this patient group with high ferritin levels could be beneficial for preventing anemia without side effects.

However, the study had some limitations. We faced some difficulty in finding patients who had received IV iron maintenance therapy because, in 2018, a policy was implemented in our center that restricted IV iron sucrose therapy, allowing it only for patients with hemoglobin levels <100 g/L. The small number of subjects and short follow-up period in this study limited the ability to adjust for other risk factors or for confounding factors on anemia. In addition, we were unable to evaluate the effect of long-term IV iron maintenance therapy on the morbidity and mortality of the patients.

## 5. Conclusions

IV iron maintenance therapy along with ESAs in children with ESRD who were undergoing regular HD was beneficial for maintaining hemoglobin levels and reducing the risk of anemia. This therapy should therefore be considered for this patient group. However, iron supplementation for patients with hyperferritinemia is a challenging issue because the high serum ferritin level may not confirm iron marker overload. Hence, the decision to start IV iron maintenance therapy in patients with hyperferritinemia should follow a holistic approach, taking into consideration the patient's clinical condition and morbidity.

## Figures and Tables

**Table 1:** Comparative baseline characteristics of the treatment group versus the comparison group.

Characteristics	Treatment group (*n* = 21)	Comparison group (*n* = 20)	*p* value
Age, mean, 95% CI	13.5 (12–15.1)	14.7 (13.8–15.7)	0.17
Male, *n* (%)	15 (71.4%)	15 (75%)	0.8
Primary disease, *n* (%)			
Urological abnormalities	3 (14.3%)	4 (20%)	
Glomerulopathy	8 (38.1%)	8 (40%)	
Kidney hypoplasia and dysplasia	10 (47.6%)	8 (40%)	
Nutritional status			
Well-nourished	8 (38.1%)	16 (76.2%)	
Malnourished	13 (61.9%)	4 (23.8%)	
Hemodialysis access			
Tunneled catheter	19 (90.5%)	18 (90%)	
Arteriovenous fistula	2 (9.5)	2 (10%)	
ESA dosage (IU/kg/week), mean, 95% CI	331.7 (256.4–407.1)	325.7 (241.1–410.3)	0.9
Hb (g/L), mean, 95% CI	125 (121–129)	131 (123–140)	0.17
MCV (fL), mean, 95% CI	87.5 (85.6–89.3)	86.2 (82.4–90.1)	0.54
MCH (pg/cell), mean, 95% CI	1.78 (28.1–29.4)	28.5 (27.2–29.9)	0.71
MCHC (g/L), mean, 95% CI	331 (325–336)	331 (323–338)	0.99
Absolute reticulocyte count (×10^9^/L), mean, 95% CI	72.9 (55.7–90)	78.1 (62.5–93.8)	0.64
Relative reticulocyte count, mean, 95% CI	0.02 (0.016–0.024)	0.019 (0.015–0.023)	0.79
SI (*µ*mol/L), mean, 95% CI	11.6 (9.5–13.7)	15.7 (12.5–18.9)	0.03
TIBC (*µ*mol/L), mean, 95% CI	35.6 (33.4–37.9)	37.3 (32.7–41.9)	0.51
TSAT (%), mean, 95% CI	34 (26.9–41.2)	43.4 (33.6–53.2)	0.11
Ferritin (*µ*g/L), mean, 95% CI	1074 (722.4–1425.6)	1799.3 (828.7–2769.9)	0.15
Dialysis vintage (days) median, range	90 (5–1014)	202 (28–927)	0.73

CI = confidence interval, ESA = erythropoiesis-stimulating agent, Hb = hemoglobin, MCV = mean corpuscular volume, MCH = mean corpuscular hemoglobin, MCHC = mean corpuscular hemoglobin concentration, SI = serum iron, TIBC = total iron binding capacity, and TSAT = transferrin saturation.

**Table 2:** Comparative characteristics of the treatment group versus the comparison group at the end of the study period.

Characteristics	Treatment group (*n* = 21)	Comparison group (*n* = 20)	*p* value
Hb (g/L), mean, 95% CI	124 (118–131)	110 (103–118)	0.005
MCV (fL), mean, 95% CI	86.4 (84.2–88.6)	86.5 (82.8–90.1)	0.96
MCH (pg/cell), mean, 95% CI,	29.7 (28.2–31.3)	28.6 (27.3–29.8)	0.22
MCHC (g/L), mean, 95% CI	336 (322–346)	479 (167–791)	0.34
Absolute reticulocytes (×10^9^/L), mean, 95% CI	80 (64.5–95.4)	78.94 (55.3–102.59)	0.94
Relative reticulocyte count, mean, 95% CI	0.02 (0.016–0.024)	0.02 (0.015–0.028)	0.71
SI (*µ*mol/L), mean, 95% CI	14.8 (11.2–18.3)	11.75 (8.2–15.3)	0.22
TIBC (*µ*mol/L), mean, 95% CI	36.8 (33.8–39.8)	36.8 (32.4–41.2)	0.99
TSAT (%), mean, 95% CI	41.8 (30.6–52.9)	34.9 (22–47.8)	0.4
Ferritin (*µ*g/L), mean, 95% CI	1310.5 (899.7–1721.2)	1313.5 (758.9–1868.1)	0.99

CI = confidence interval, Hb = hemoglobin, MCV = mean corpuscular volume, MCH = mean corpuscular hemoglobin, MCHC = mean corpuscular hemoglobin concentration, SI = serum iron, TIBC = total iron binding capacity, and TSAT = transferrin saturation.

## Data Availability

The data used to support the findings of this study are available from the corresponding author upon request.
